# Factors Contributing to the Delayed Vaccination Among Children in Riyadh City, Saudi Arabia: A Cross-Sectional Study

**DOI:** 10.7759/cureus.43188

**Published:** 2023-08-09

**Authors:** Mooj A Alghofaili, Sultan O Aljuaid, Najd Alqahtani, Muwred Alghufaili, Eman E Abd-Ellatif

**Affiliations:** 1 Preventive Medicine, Ministry of Health, Riyadh, SAU; 2 Preventive Medicine, Prince Sultan Military Medical City, Riyadh, SAU; 3 College of Medicine, Princess Nourah Bint Abdul Rahman University, Riyadh, SAU; 4 Linguistics, Ministry of Education, Riyadh, SAU; 5 Public Health and Preventive Medicine, Mansoura University, Mansoura, EGY

**Keywords:** vaccine timeliness, vaccine coverage, vaccination prevalence, child vaccination, delayed vaccination, vaccination

## Abstract

Background

Immunizations protect children from deadly infectious diseases. The timeliness of vaccinating children is crucial to ensure effective immunization and to decrease the burden of many infectious diseases. Therefore, this study assessed the prevalence and determinants of vaccination delay among children in Riyadh City, Saudi Arabia.

Methods

This cross-sectional study was conducted at the primary healthcare centers in Riyadh City, Saudi Arabia, on 593 parents with children of two years of age or below. It used a self-administered questionnaire inquiring about socio-demographic characteristics and assessing the vaccination statuses of their children and the causes of delayed vaccinations.

Results

The results showed that 7.1% of children had a delay in the previous vaccination. Of those delays, collectively, 77.5% were delays in inactivated poliovirus vaccine (IPV), oral poliovirus vaccine (OPV), and meningococcal vaccine (MCV) vaccines. The delay was mostly caused by an illness of the child on vaccination day, carelessness of parents, or long postponement. After adjusting for confounders, the father's high school or bachelor's education level (OR = 1.18, 95% CI: 1.03, 1.36) (p<0.05), child's mix-type nutrition (OR = 1.06, 95% CI: 1.02, 1.10) (p=0.001), and the belief that multiple vaccines are harmful to the child (POR = 1.03, 95% CI: 1.01, 1.06) (p=0.005) were positively associated with vaccination delay, while prematurity was negatively associated with vaccination delay (OR = 0.96, 95% CI: 0.93, 0.99) (p=0.031).

Conclusion

The study found the prevalence of vaccination delay was lower than in previous COVID-19-era studies. The child's illness was the main reason for the delay. Factors like parental education, nutrition type, and vaccine beliefs contributed to delays, while prematurity reduced delays. Measures should be strengthened to increase vaccination coverage for children.

## Introduction

It is estimated that over 2.5 million deaths are avoided through vaccination every year, and according to the WHO, around 19.5 million infants worldwide, unfortunately, do not get basic vaccines [1-3] despite the Expanded Program on Immunization, which aimed to reach 90% vaccination coverage in every country around the world that was initiated in 1974 by the WHO [2].

In the Kingdom of Saudi Arabia, immunization started with diphtheria, tetanus, and pertussis (DTP) vaccination in 1979, and it was later expanded to include additional vaccines. Vaccination is considered a prerequisite for school entry at the age of six years in Saudi Arabia [1]. However, according to the WHO, the vaccination coverage rate for those in Saudi Arabia was 52% for Bacille Calmette-Guerin (BCG) compared to 98% in 2018, and the coverage rates for DTP and measles, mumps, and rubella vaccine (MMR) were 98% and 96%, respectively. Although this is a high immunization rate, the number of reported cases in 2019 was 1035 for measles, 187 for mumps, 326 for pertussis, and 62 for rubella [2]. The Saudi National Immunization Program recommends many vaccines to be administered in the first 24 months of life, including hepatitis B vaccine at birth, pneumococcal conjugate, rotavirus, inactivated poliovirus vaccine (IPV), meningococcal vaccine (MCV), diphtheria, tetanus, polio, *Haemophilus influenzae* type b (HIB) vaccines, BCG, meningococcal vaccine, varicella, MMR, and hepatitis A [3]. Vaccination programs are monitored by timely vaccination. Timely vaccination ensures optimum protection of the children. However, vaccine delay, defined as 30 days or more after the recommended age for each dose [4,5], could affect the subsequent doses, increasing the child’s risk of disease [6,7].

Vaccine acceptance and practice are affected by factors such as being unwilling to vaccinate among well-educated upper and middle-income people, which is related to health beliefs [8]. Socioeconomic or religious factors can contribute to delays. It was found that children with less educated mothers, children within larger households, and those who belong to minority groups and immigrants were less likely to receive their vaccination on time [9-11]. Additionally, the misbelief that vaccines can cause autism and disabilities among parents in Saudi Arabia was also reported to affect timely vaccination [12].

The COVID-19 pandemic has also affected immunization systems and schedules worldwide [13]. Three-quarters of countries worldwide, including Saudi Arabia, reported the disruption disruptions in their immunization programs as a result of the SARS-CoV-2 pandemic, including limited movements in lockdowns, transport interruptions, economic hardships, fear of COVID-19 exposed exposure, and insufficient healthcare staff due to redeployment to COVID-19 response duties [14]. Studies conducted in Saudi Arabia found that nearly 73.2% of parents made an appointment for their child's vaccination during the pandemic. However, around 23.4% of parents reported that their child's vaccination was delayed by over a month. Studies also demonstrated the increase in vaccination hesitancy following the COVID-19 pandemic and its vaccination campaign [15,16].

These reported challenges highlight the urgent need to explore further the vaccination program in Saudi Arabia to inform policies and programs designed to get back on track and ensure full coverage for all children in Saudi Arabia. Post-pandemic studies are essential to evaluate the vaccination delays and contributing factors in order to inform health authorities, public health professionals, and partners in Saudi Arabia in their efforts to streamline successful vaccination strategies. Therefore, we conducted this post-pandemic study to assess the prevalence and determinants of vaccination delay in children aged two years or less in Riyadh City, Saudi Arabia.

## Materials and methods

Study design and setting

We conducted an analytical cross-sectional study between January and March 2023. We targeted Saudi parents of healthy children aged two years or less attending the PHCCs and vaccination in Riyadh City who had vaccination cards.

Parents who were non-Saudi, who were not accompanied by their children, and who had sick children were excluded.

Sample size and sampling method

Assuming a 95% confidence interval, 5% margin of error, and response distribution of 50% with a design effect of 1.5, the estimated minimum sample size was 566. By using a convenient sampling technique, we selected 593 eligible participants from PHCCs.

Data collection tool and technique

Data collection was done using a self-administered questionnaire in Arabic language. The questionnaire consisted of two parts: the first part included child characteristics, parental information, the child’s physical well-being, socioeconomic status, number of delayed vaccinations, duration and reasons for vaccine delays, and the second part assessed parents’ awareness. Data on vaccination timing were collected from vaccination cards.

The questionnaire was prepared by the authors, reviewed for validity by three consultants in family and community medicine, and then pre-tested for comprehension on 15 participants who were not included in the study. The sample has been taken from Riyadh's second health cluster, which has 40 PHCCs; a random sample of 10 PHCCs has been selected with an average of 60 participants from each center. Then, the investigators visited each PHCC and invited the parents to participate in the study during the waiting time after explaining the objectives of the study to them. The questionnaire sheets were then handed over to the parents, who consented to fill them, and they were immediately collected afterward.

Data management and statistical analysis

Data entry and analysis were done using SPSS Statistics version 23 (IBM Corp. Released 2015. IBM SPSS Statistics for Windows, Version 23.0. Armonk, NY: IBM Corp.). We performed descriptive statistics and presented data as frequencies, percentages, or median (IQR). Regression analyses were performed to identify associations between the prevalence of vaccination delays and socio-demographic characteristics. The OR and CI were calculated, and a statistical significance was considered when the p-value was less than 0.05.

Ethical considerations

Every participant was informed about the purpose of the study, written consent to participate was obtained, and they were allowed to withdraw at any time. The questionnaire was anonymous to ensure confidentiality, and no private questions were included. The data collected were never disclosed to anyone outside this study. Ethical approval was obtained from the King Fahad Medical City Institutional Review Board (Ref. No: 23-050E).

## Results

The majority of participants were aged 26-35 (53%), followed by 36-45 years old (25%). Most participants had 18- to 24-month-old children (20%), followed by 9 to 12 months old (18%). Regarding the sex of the children, it was found that most of them were female (53%), and the rest were male. The majority of mothers had bachelor's degrees (44%), followed by those with high school certificates (39%), and most mothers were employed (57%). The majority of fathers were aged 36-45 (54%), followed by those who were 26-35 years old (33%). Like mothers, most fathers had bachelor’s degrees (61%), followed by high school certificates (22%), and 97% of fathers were employed. Most families had an income of more than 10,000 Saudi Rials (SR) (46%), followed by families with 5000-10,000 SR income (32%), and 61% of the families had two to four children. Table 1 gives further details of the participants.

**Table 1:** Background characteristics of the children SR: Saudi Rials

Characteristics	n (%)
Age of the child	
Birth to 2 months	48 (8.1)
2 to 4 months	73 (12)
4 to 6 months	70 (12)
6 to 9 months	88 (15)
9 to 12 months	104 (18)
12 to 18 months	93 (16)
18 to 24 months	117 (20)
Sex of the child	
Female	315 (53)
Male	278 (47)
Order of birth	
First	181 (31)
Second	212 (36)
Third	129 (22)
Fourth or more	71 (12)
Mother’s age	
<= 25 years old	103 (17)
26-35 years old	317 (53)
36-45 years old	151 (25)
>= 46 years old	22 (3.7)
Mother’s education	
Not educated	8 (1.3)
Below high school	49 (8.3)
High school	230 (39)
Bachelor’s degree	259 (44)
Above bachelor’s degree	47 (7.9)
Mother’s employment	
Employed	336 (57)
Not employed	257 (43)
Father’s age	
<=25 years old	11 (1.9)
26-35 years old	197 (33)
36-45 years old	317 (54)
>46 years old	67 (11)
Unknown	1
Father’s education	
Not educated	0 (0)
Below high school	17 (2.9)
High school	130 (22)
Bachelor’s degree	363 (61)
Above bachelor’s degree	83 (14)
Father’s employment	
Employed	574 (97)
Not employed	19 (3.2)
Family income	
Less than 5000 SR	40 (6.7)
Between 5000-10000 SR	189 (32)
More than 10000 to 25000 SR	272 (46)
More than 25000 SR	92 (16)
Number of children in the family	
Only one child	182 (31)
2-4 children	359 (61)
5 or more children	52 (8.8)

As shown in Table 2, most children were born at term (81%), with a birth weight of 2.5-3.5 kg (63%). The nutrition intake was mostly the combination of formula and breastfeeding (42%), followed by exclusive breastfeeding (21%). Most children were healthy (97%), and of those sick, 4.2% suffered from allergies. Of those allergic, most had allergies to strawberries (33.1$%) and eggs (22.2%). Of children with chronic diseases (1.9%), 44.4% had asthma, and 33.3% had Down syndrome. Only 2% had been vaccinated in the past four weeks, and 0.5% had serious reactions to the past vaccination.

**Table 2:** Health and nutritional status of the children G6PD: glucose-6-phosphate dehydrogenase, Kg: kilogram

Characteristics	n (%)
Is the child sick today?	N=593
No	575 (97.0)
yes	18 (3.0)
Does the child have allergies to medications, food?	N=593
No	568 (95.8)
yes	25 (4.2)
Child is allergic to	N= 9
Chocolate	1 (11.1)
Egg	2 (22.2)
Nuts	1 (11.1)
G6PD	1 (11.1)
Strawberry	3 (33.1)
Ants	1 (11.1)
Has the child had a serious reaction to a vaccine in the past?	N=593
No	590 (99.5)
yes	3 (0.5)
Has the child had a health problem or chronic disease?	N=593
No	582 (98.1)
yes	11 (1.9)
Health problems of the child	N=9
Asthma	4 (44.4)
Diabetes	1 (11.1)
Down syndrome	3 (33.3)
Eggs allergies	1 (11.1)
Has the child received vaccinations in the past 4 weeks?	N=593
No	581 (98.0)
yes	12 (2.0)
Is the child on current medication?	N=593
No	588 (99.2)
yes	5 (0.8)
Child’s gestational age at delivery	N=593
Full-term	480 (81)
Premature	113 (19)
Child’s birth weight	N=593
<1.5 kg	0 (0)
<2.5 kg	156 (28)
2.5-3.5 kg	352 (63)
>3.5 kg	47 (8.5)
Child’s current nutrition intake	N=593
Breastfeeding	123 (21)
Mix	249 (42)
Formula feed	221 (37)

As seen in Table 3, 6.2% of parents believed their children's vaccination were delayed, with a median (IQR) delay of 14 (6,56) days. Previous delays in vaccination were reported by 7.1%. Most delayed vaccines were inactivated poliovirus vaccine (IPV), oral poliovirus vaccine (OPV), and meningococcal vaccine (MCV), followed by DTP, hepatitis B, and HIB. The delay was mostly caused by an illness of the child on vaccination day (66.1%), followed by negligence (16.1%).

**Table 3:** Vaccination details of the children IQR: interquartile range, BCG: Bacille Calmette-Guerin, DTP: diphtheria, tetanus, and pertussis, HIB: hemophilus Influenza type b, IPV: inactivated poliovirus vaccine, MCV: meningococcal vaccine, MMR: measles, mumps, and rubella, OPV: oral poliovirus vaccine

Characteristics	n (%)
Parent thinks that the child’s vaccination is delayed	N=593
I don’t know	74 (12.5)
No	482 (81.3)
Yes	37 (6.2)
Duration of delay (in days), median (IQR)	14 (6, 56)
Does he have previous delay in his\her schedule?	N=593
No	551 (92.9)
Yes	42 (7.1)
Vaccine delay	N=19
Delay in all vaccines	7 (1.2)
Delays in some vaccines	12 (2.0)
Vaccines that were delayed (multiple responses)	N= 61
BCG	1 (1.6)
DTP	5 (8.2)
Hepatitis A	1(1.6)
Hepatitis B	4 (6.6)
HIB	4 (6.6)
IPV	19 (31.1)
MCV	10 (16.4)
MMR	3 (4.9)
OPV	14 (23.0)
Reasons for vaccination delay	N= 56
No appointment available	1 (1.8)
Negligence (not given)	9 (16.1)
Other reasons	2 (3.6)
Delayed by the time of appointment	3 (5.4)
Fear from side effect	1 (1.8)
Illness of the child at the vaccination day	37 (66.1)
Unavailability of the vaccine	2 (3.6)
Very far center/difficult transportation	1 (1.8)

Regarding vaccination awareness, 58.3% of participants know that they prevent infections, 18.6% think vaccination is just for following protocols, and 18.6% vaccinate to complete the table to attend school. Some participants believed there was a link between the MMR vaccine and autism (15.5%), while 39.5% did not know. Most side effects reported were fever (44.4%), autism (7.4%), pain (3.7%.), and rash (3.7%.). However, 33.3% could not identify any side effects. Most (66.9%) thought that children should be vaccinated as scheduled. When the vaccine is unavailable, most participants reported trying to find it in another center or clinic (60.7%), followed by those who returned a month later (23.6%). Within 48 hours post-vaccination, most participants (41%) reported giving paracetamol to children with fever, while others used cold compression (32.6%) and called doctors to ask (18.8%). Over half (53.1%) of participants did not know the impact of the COVID-19 pandemic on their decision, and 29.5% thought that giving multiple vaccines is harmful to the child. Table 4 shows participants' awareness of childhood vaccination.

**Table 4:** TABLE 4: Parent’s awareness of childhood vaccination

Characteristics	n (%)
Reason for thinking why vaccinations are important	N=593
Prevent infections	486 (81.9)
It’s just protocols to follow	155 (26.2)
To complete the table to attend school	152 (25.6)
I don’t know	40 (4.8)
I still believe there could be a link between the MMR vaccination and autism	N=593
I don’t know	234 (39.5)
No	267 (45.0)
Yes	92 (15.5)
I worry about the possible side effects of vaccinations. If yes, explain which symptoms	N=593
I don’t know	209 (35.2)
No	333 (56.2)
Yes	51 (8.6)
Possible side effects of vaccination	N=54
Fever	24 (44.4)
Don't know	18 (33.3)
Autism	4 (7.4)
Pain	2 (3.7)
Rash	2 (3.7)
Face swelling	1 (1.9)
Heart problems	1 (1.9)
Nausea	1 (1.9)
Swelling	1 (1.9)
Do you think that your kid needs to take the vaccine as scheduled?	N=593
I don’t know	119 (20.1)
No	77(13)
Yes	397 (66.9)
What do they do when a vaccine is not available?	N=593
Try to find it in another center or clinic to give him on time	360 (60.7)
You come back after a month to check	140 (23.6)
Wait until the next vaccination date	93 (15.7)
What they do at home 48 hours after vaccination	N=812
Paracetamol to avoid fever	333 (41.0)
Cold compression on the injection site	265 (32.6)
Call your doctor for any concerns	153 (18.8)
Nothing to do	52 (6.4)
Others	9 (1.1)
Impact of COVID-19 pandemic on decision to vaccinate	N=593
I don’t know	315 (53.1)
Negative	80 (13.5)
Positive	198 (33.4)
Do you think giving multiple vaccines at the same visit is harmful to your child?	
I don’t know	261 (44.0)
No	157 (26.5)
Yes	175 (29.5)

The univariate regression analysis showed that parents’ education, family income, child’s gestational age at delivery, child’s current nutritional intake, and parent’s belief that giving multiple vaccines was harmful to the child were significantly associated (p<0.05) with vaccination delay (Table 5).

**Table 5:** Factors associated with vaccination delay SR: Saudi Rials, MMR: measles, mumps, and rubella vaccine, P<0.05: statistically significant

Characteristics	Overall N = 593 n (%)	Delayed N = 52 n (%)	No delay N = 541 n (%)	p-value
Age of the child				0.057
>9 months	314 (100)	21 (6.7)	293 (93)	
≤9 months	279 (100)	31 (11)	248 (89)	
Sex of the child				0.3
Female	315 (100)	31 (9.8)	284 (90)	
Male	278 (100)	21 (7.6)	257 (92)	
Order of birth				>0.9
First	181 (100)	15 (8.3)	166 (92)	
Second	212 (100)	21 (9.9)	191 (90)	
Third	129 (100)	10 (7.8)	119 (92)	
Fourth or more	71 (100)	6 (8.5)	65 (92)	
Mother’s age				0.4
≤25 years old	103 (100)	7 (6.8)	96 (93)	
26-35 years old	317 (100)	34 (11)	283 (89)	
36-45 years old	151 (100)	10 (6.6)	141 (93)	
≥46 years old	22 (100)	1 (4.5)	21 (95)	
Mother’s education				<0.001
Not educated	8 (100)	2 (25)	6 (75)	
Below high school	49 (100)	15 (31)	34 (69)	
High school	230 (100)	22 (9.6)	208 (90)	
Bachelor’s degree	259 (100)	11 (4.2)	248 (96)	
Above bachelor’s degree	47 (100)	2 (4.3)	45 (96)	
Mother’s employment				0.2
Employed	336 (100)	25 (7.4)	311 (93)	
Not employed	257 (100)	27 (11)	230 (89)	
Father’s age				0.6
≤25 years old	11 (100)	1 (9.1)	10 (91)	
26-35 years old	197 (100)	18 (9.1)	179 (91)	
36-45 years old	317 (100)	30 (9.5)	287 (91)	
≥46 years old	67 (100)	3 (4.5)	64 (96)	
Father’s education				<0.001
Below high school	17 (100)	7 (41)	10 (59)	
High school	130 (100)	16 (12)	114 (88)	
Bachelor’s degree	363 (100)	23 (6.3)	340 (94)	
Above bachelor’s degree	83 (100)	6 (7.2)	77 (93)	
Father’s employment				0.076
Employed	574 (100)	48 (8.4)	526 (92)	
Not employed	19 (100)	4 (21)	15 (79)	
Family income				<0.001
Less than 5000 SR	40 (100)	6 (15)	34 (85)	
Between 5000-10000 SR	189 (100)	30 (16)	159 (84)	
More than 10000 to 25000 SR	272 (100)	11 (4.0)	261 (96)	
More than 25000 SR	92 (100)	5 (5.4)	87 (95)	
Number of children in a family				0.5
Only one child	182 (100)	16 (8.8)	166 (91)	
Two to four children	359 (100)	34 (9.5)	325 (91)	
Five or more children	52 (100)	2 (3.8)	50 (96)	
Child has allergies				0.059
No	568 (100)	47 (8.3)	521 (92)	
yes	25 (100)	5 (20)	20 (80)	
The child has health problems or chronic diseases				0.2
No	582 (100)	50 (8.6)	532 (91)	
yes	11 (100)	2 (18)	9 (82)	
Child’s gestational age at delivery				0.024
Full term delivery	480 (100)	36 (7.5)	444 (92)	
Premature delivery	113 (100)	16 (14)	97 (86)	
Child’s birth weight				0.8
<2.5 kg	156 (100)	13 (8.3)	143 (92)	
2.5-3.5 kg	352 (100)	33 (9.4)	319 (91)	
>3.5 kg	47 (100)	5 (11)	42 (89)	
Child’s current nutrition intake				0.001
Breastfeeding	123 (100)	18 (15)	105 (85)	
Mix	249 (100)	10 (4.0)	239 (96)	
Formula feed	221 (100)	24 (11)	197 (89)	
Believes there is a link between the MMR vaccine and autism				0.2
No/don't know	501 (100)	47 (9.4)	454 (91)	
Yes	92 (100)	5 (5.4)	87 (95)	
Worries about the side effects of the vaccine				0.8
No/don't know	542 (100)	47 (8.7)	495 (91)	
Yes	51 (100)	5 (9.8)	46 (90)	
Belief that giving multiple vaccines is harmful to the child				0.008
No/don't know	418 (100)	45 (11)	373 (89)	
Yes	175 (100)	7 (4.0)	168 (96)	

Multivariate regression analysis showed that fathers having high school or bachelor’s level education (POR = 1.18, 95% CI: 1.03, 1.36), children receiving mix-type of nutrition (OR = 1.06, 95% CI: 1.02, 1.10), and parents thinking that giving multiple vaccines was harmful to the child (OR = 1.03, 95% CI: 1.01, 1.06) were positively associated with vaccination delay, whereas a child born prematurely (OR = 0.96, 95% CI: 0.93, 0.99) was negatively associated with vaccination delay (Table 6).

**Table 6:** Independent factors associated with vaccine delay SR: Saudi Rials, CI: confidence interval, P<0.005: statistically significant

	Adjusted odds ratio			p-value
	Estimate	95% CI		
(Intercept)	1.55	1.29	1.87	<0.001
Age of the child				
>9 months	1			
≤9 months	0.98	0.96	1.01	0.162
Mother’s education				
Not educated	1			
Below high school	0.95	0.82	1.09	0.438
High school	1.06	0.93	1.2	0.377
Bachelor’s degree	1.08	0.95	1.22	0.26
Above bachelor’s degree	1.1	0.97	1.26	0.142
Father’s education				
Below high school	1			
High school	1.18	1.03	1.36	0.015
Bachelor’s degree	1.18	1.03	1.35	0.018
Above bachelor’s degree	1.15	1	1.32	0.053
Father’s employment				
Employed	1			
Not employed	0.95	0.86	1.05	0.326
Family income				
Less than 5000 SR	1			
Between 5000-10000 SR	0.94	0.88	1.01	0.079
More than 10000 to 25000 SR	0.98	0.92	1.05	0.607
More than 25000 SR	0.97	0.9	1.04	0.414
Child has allergies	0.94	0.87	1.02	0.114
Child was delivered prematurely	0.96	0.93	0.99	0.031
Baby nutrition				
Breastfeeding	1			
Mix	1.06	1.02	1.1	0.001
Formula feed	1.03	0.99	1.07	0.203
Think giving multiple vaccines is harmful to the child	1.03	1.01	1.06	0.005

## Discussion

Childhood vaccination is one of the most beneficial public health strategies to keep diseases under control. Vaccination delay for children can have significant consequences on their health and well-being, with some potential impacts including high risks of diseases and reduced herd immunity, which can lead to serious complications, hospitalization, and mortality [17,18]. This study evaluated the prevalence of vaccination delay among children in Saudi Arabia and associated determinants. As it is a post-pandemic study, it will help to inform about the performance of immunization programs in Riyadh after the pandemic that had affected the programs leading to reduced routine vaccination.

This study revealed that 6.2% and 7.1% of parents reported that their children had not received vaccinations on time during this study period and in the past, respectively, which was lower than 9% reported by a previous study conducted at the King Abdulaziz University Hospital [19]. Another study conducted at five PHCCs and two tertiary governmental hospitals in Jeddah reported that 24.2% of the children had delayed vaccination, which is more than three times our reported prevalence [17]. However, in 2009, the National Immunization Survey involving 11,206 children reported a higher rate of delayed vaccination (39.8%) [20]. A study conducted in India also reported that children between the ages of 10 and 23 months had delayed receiving the BCG (23.1%), DTP-first dose (29.3%), and measles vaccines (34.8%), which are higher than or similar to other studies conducted in Saudi Arabia but lower than what our study revealed [17,20,21]. This Indian study showed that children from Muslim families were more likely to delay getting a vaccination, which may explain the prevalence reported by aforementioned studies in Saudi Arabia, a Muslim country. However, our study showed that the main reason for the delay was the illness of the child on vaccination day. Similarly, a previous study in Al-Madinah Al-Munawarah found that signs of sickness in children were the main reason for delayed vaccination among 42.2% of children [21]. In contrast, a previous study conducted in Jeddah showed that 21.3% of delayed vaccination cases were due to traveling difficulties [17]. Similarly, a systematic review of studies published between 1999 and 2009 showed that the most common reasons for the delay were transportation problems, negligence, late birth orders, difficulties with appointments, upper respiratory tract illnesses, physicians’ advice to delay, and forgetting the vaccination schedules [22]. Studies found that the child's age was a statistically significant determinant of complete vaccination (p = 0.012) in Kenya [23]. Similarly, a study conducted in Athens, Greece, concluded that the child’s age was strongly associated with incomplete vaccination for all vaccines (p <0.001) [24]. These studies align with our findings that a child’s gestational age at delivery was significantly associated with vaccination delay. However, age was not significant after adjusting for confounders. On the other hand, we found that the father’s education level, the child receiving a mix-type of nutrition, and the parent thinking that giving multiple vaccines was harmful to the child were independent determinants of vaccination delay.

The low birth weight associated with low gestation age is a known cause of vaccination delay, especially among premature children and those below 2000 g. This is attributed to parental concern about the safety and benefit of vaccination [25]. Studies reported that early age at first childbirth and lower maternal education was associated with delayed vaccination [26]. However, we could not find a significant association between parental age and vaccination delay. Consistent with our study, studies reported that a high maternal level of education was linked to vaccine hesitancy, leading to vaccine delays [27,28]. This is also similar to what was reported by an Iranian study that birth order and the mother’s educational level were the major factors for delayed vaccination [29].

The COVID-19 pandemic has affected normal schedules of immunization which might have led to a high prevalence (23-51%) of delayed vaccines reported in Saudi Arabia by previous studies [30,31]. This is supported by a study that found 60% of parents reporting delayed vaccination for their children during the pandemic as they did not attend appointments on time due to fear of contracting COVID-19 [30]. The fact that our study showed a lower vaccination delay rate (7.1%) may indicate that post-pandemic, most factors contributing to the delay have been removed since the lockdowns were lifted, COVID-19 infections dropped significantly, and Saudi Arabia opened up again as it was in the pre-pandemic era. This also signals the continued recovery of health systems and immunization programs. Our findings contrast the report of the WHO signaling the unexpected continuing decline in routine vaccine uptake post-pandemic. The WHO reported that routine vaccination coverage has decreased during and even after the pandemic, affecting up to 25 million children [32]. Some reasons for the decrease are the COVID-19 pandemic's impact on health systems and the distribution of COVID-19 vaccines, supply chain problems due to lockdowns, limited human resources, and financing constraints. Increased vaccine misunderstanding, skepticism, and hesitancy following the pandemic have also been identified as contributing factors [13]. Though our study showed that most participants did not know the impact of the COVID-19 pandemic on their child’s vaccination, misbelief that some vaccines are associated with autism was found, and the misbelief that giving children multiple vaccines is harmful was the independent factor associated with vaccine delay. We found that prematurity was associated with lower vaccination delay prevalence. This might be explained by the fact that parents of premature babies tend to be educated and extra careful to ensure their vulnerable babies are fully protected from all possible diseases. This normally contrasts with evidence that prematurity leads to vaccination delays [33], as premature babies are more likely to be unfit for vaccination due to health issues.

There are some limitations to the study. For instance, using a convenience sample limits the generalizability of the findings to the entire population. Moreover, using a cross-sectional study design does not permit concluding the causality of the emergent predictive relationship, and the self-reported questionnaire is prone to over- and under-reporting that may affect the interpretation of results. This study was also conducted in a single city, which might not reflect the situation in other cities and rural areas of Saudi Arabia. We recommend more extensive studies encompassing nationwide samples with prospective designs to mitigate these limitations and show vaccination coverage trends in the future.

## Conclusions

The findings showed that the prevalence of vaccination delay was lower than what previous studies reported, most of which were conducted during the COVID-19 pandemic, indicating an improvement in vaccination coverage, especially post-pandemic. The most common reason for the delay was reported to be a child’s illness. However, the significant independent determinants, such as parental level of education, child’s nutrition type, and parent thinking that giving multiple vaccines was harmful to the child, contributed to more vaccination delays, while prematurity reduced the likelihood of delays. Therefore, the study's results show that vaccine delay prevalence was low, but measures were strengthened to keep increasing the coverage for all children.
